# Supplementary material for: Tinamiphilopsis temmincki sp. n., a New Quill Mite Species from Tataupa Tinamou, and the Early History of Syringophilid Mites
Source: Animals (Basel). 2023 Aug 28;13(17):2728. doi: 10.3390/ani13172728 (PMC10486729; doi:10.3390/ani13172728)
Supplement: Supplementary file 1 [file animals-13-02728-s001.zip › animals-2514070-supplementary.pdf]

**Supplementary Table S1.** List of characters used in the phylogenetic analysis of the primitive syringophilid genera (genera with a full complement of body and leg setae).

| No. | Character |
|---|---|
| 1 | Basal part of gnathosoma: not submerged (0); deeply submerged into idiosoma (1) |
| 2 | Stylophore apodeme: indistinct (0); distinctly developed (1) |
| 3 | Palpal tibia and tarsus: separated (0); fused (1) |
| 4 | Claw-like seta of palps: present (0); absent (1) |
| 5 | Posterior part of stylophore: constricted (0); rounded (1) |
| 6 | Projection on posterior part of stylophore: absent (0); present (1) |
| 7 | Hypostomal apex: unornamented (0); ornamented (1) |
| 8 | Hypostomal protuberances: one pair small and blunt-ended (0); one pair small and sharp-ended (1); two pairs (2); one pair large (3) |
| 9 | Size of hypostomal lips: small (0); large (1) |
| 10 | Hypostomal lips: equal (0); unequal (1) |
| 11 | Lateral branch of peritreme: normal i.e., 6-10 chambers (0); reduced i.e, 1-5 chambers (1); supernumerary i.e, 11-20 chambers (2) |
| 12 | Distal tip of chelicerae: edentate (0); dentate (1) |
| 13 | Propodonotal shield: strongly sclerotized (0); weakly sclerotized (1) |
| 14 | Hysteronotal shield: present (0); absent (1) |
| 15 | Hysteronotal shield: entire (0); divided (1) |
| 16 | Hysteronotal shield: reach level of setae *d2* (0); not reach *d2* (1) |
| 17 | Pygidial shield: present (0); absent (1) |
| 18 | Hysteronotal and pygidial shields: fused (0); not fused (1) |
| 19 | Position of setae *vi* and *ve*: at same level (0); *vi* anterior to *ve* (1) |
| 20 | Position of setae *c1* and *se*: at same level (0); not at same level (1) |
| 21 | Position of setae *se* and *c2*: *se* posterior to *c2* (0); *se* and *c2* at same level (1) |
| 22 | Position of setae *c2*: out of shield (0); on shield (1) |
| 23 | Ornamentation of idiosomal setae: present (0); absent (1) |
| 24 | Setae *e1*: present (0); absent (1) |
| 25 | Position of setae *f1* and *f2*:*f1* close to *f2* (0); *f2* far from *f1* (1) |
| 26 | Setae *ps3*: present (0); absent (1) |
| 27 | Genital setae: short (0); long (1); medium (2) |
| 28 | Position of setae *ag1* and *ag2*: *ag2* postero lateral to *ag1* (0); *ag2* posterior to *ag2* (1); *ag1* and *ag2* at same level (2) |
| 29 | Neotrichious aggenital setae: absent (0); present (1) |
| 30 | Seta a' on tarsus I: present (0); absent (1) |
| 31 | Proral setae *p'* and *p"*: rod-like (0); fan-like (1) |
| 32 | Number of tines in proral setae: reduced i.e, 4-10 tines (0); normal i.e, 11-20 tines (1); supernumerary 21-35 tines (2) |
| 33 | Setae *4a*: present (0); absent (1) |
| 34 | Seta *vs* on tarsus III: present (0); absent (1) |
| 35 | Seta *vs* on tarsus IV: present (0); absent (1) |
| 36 | Seta *dG* on genu IV: present (0); absent (1) |
| 37 | Seta *vF* on femur III: present (0); absent (1) |
| 38 | Seta *v* on trochanter III: present (0); absent (1) |
| 39 | Legs I and II: subequal in thickness (0); I thicker than II (1) |
| 40 | Legs I and II: I longer than II (0); I and II subequal in length (1) |
| 41 | Coxal fields I-II and III-IV: grouped together (0); widely separated (1) |
| 42 | Apodemes I and II: similar in shape (0); different in shape (1) |
| 43 | Apodemes I: divergent (0); parallel (1) |
| 44 | Divergence of apodemes I: strongly divergent (0); slightly divergent (1) |
| 45 | Apodemes I and II: fused (0); not fused (1) |
| 46 | Apodemes III and IV: present (0); absent (1) |
| 47 | Claw basal angle: absent (0); present (1) |
| 48 | Position of setae *3a* and *3b*: at same level (0); *3a* anterior to *3b* (1) |
| 49 | Body size: large i.e., more than 1000 (0); medium i.e., 700-990 (1); small i.e., less than 690 (2) |

**Supplementary Table S2.** Data matrix

| | 1 2 3 4 |
|---|---|
| | 1234567890123456789012345678901234567890123456789 |
| Cheyletus eruditus | 0000--0-00000000010000100000010-00000000000010000 |
| Cheletopsis norneri | 0000--0-000001--1-0101100000010-00000000000010000 |
| Aulobia dendroicae | 1111100-11200001011000010100001011111101111-11011 |
| Aulobia cardueli | 1111100-11200001011000010100001011111101111-11011 |
| Blaszakia rossae | 1111100-000000000112001111010010111111111110111012 |
| Bubophilus aegolius | 1111000-0011000101100001010100111111110111011 1011 |
| Charadriphilus ludmilae | 1111000-002000000011200111101001011111101110111012 |
| Charadriphilus ralli | 1111000-010000000011210111101001011111101110111012 |
| Colinophilus wilsoni | 1111000-002100000011200010101001111111111110111010 |
| Corvitorotroglus alpha | 1111001100010001011101010 10-1011111111101110001010 |
| Creagonycha totani | 1111000-000111--0-1101010101001111111101100011000 |
| Crotophagisyringophilus io | 11110011002101--0-1102010101001111111101110101010 |
| Ixobrychiphilus wallacei | 1111000-0011100100110001010100101111110111 1-11012 |
| Kalamotrypetes cracidus | 1111110-000000000011200010100001011111111110111012 |
| Megasyringophilus eos | 11110012001101--1-1101011100001111111101110010100 |
| Megasyringophilus trichoglossus | 1111001?0001000101110201111000111111110111001 0100 |
| Megasyringophilus aquilus | 11110011000100010111020111000012111111011100 10100 |
| Pteroclidisyringophilus otididus | 1111000-001000010113000101000011111111011101 11012 |
| Pteroclidisyringophilus re | 1111000-00100000001121001010000111111110111011 1012 |
| Selenonycha charadriiformicus | 1111101-0001000101110101110100111111110110001 0000 |
| Syringophilpsis turdi | 11110012002101--0-1101010102001011111101110001010 |
| Syringophilopsis muscicapicus | 1111001200210011001101010101001011111101110001010 |
| Syringophilus bipectinatus | 1111000-000000000113100101000011111111011101 01011 |
| Tinamiphilopsis ariconte | 1111010-000100000011300010101001111111101110111012 |
| Tinamiphilopsis elegans | 1111010-000100000011300010101001111111101110111012 |
| Tinamiphilopsis temmincki | 1111010-000100000011300010101001111111101110111012 |
| Torotrogla meulae | 1111001300010011001101010 10-1011111111101100011011 |
| Torotrogla lusciniae | 11110013000101--0-110101010-1011111111101100010011 |
| Trypetoptila casmerodia | 1111011???0101--0-100001011-1012101111 01110001010 |
